# The comprehensive double loop activities for patient safety management

**DOI:** 10.1016/j.amsu.2022.103520

**Published:** 2022-04-01

**Authors:** Tatsuya Fukami, Yoshimasa Nagao

**Affiliations:** Department of Patient Safety, Nagoya University Hospital, 65 Tsurumai-cho, Showa-ku, Nagoya, 466-8560, Aichi, Japan

**Keywords:** Incident report, Response to the critical situation, Quality improvement, Safety management

## Abstract

We practice patient safety as a model that links patient safety and quality improvement in healthcare. The most important activity is the incident report. The loop on the left is during usual situation activity related to quality improvement in healthcare. The loop on the right is during critical situations activity related to patient safety. What is important in these activities is the initial response to the critical situation, which is the first corner of the right loop. We practice emphasizing the initial response to the critical situation, creating the pattern, and taking measures without omissions. Although many patient safety measures have been taken, it has become clear that there is a shortage of doctors who can practice them. We have practiced that pattern and supported advanced healthcare. We want you to explain the pattern and use it in practice.

## Highlights

• Patient safety managers need to recognize the overall picture of patient safety.
• Divided patient safety management into usual and critical situations, illustrated it as loops.
• Facilitate to recognize how to approach the daily issues for patient safety managers.

**Fig. 1:**
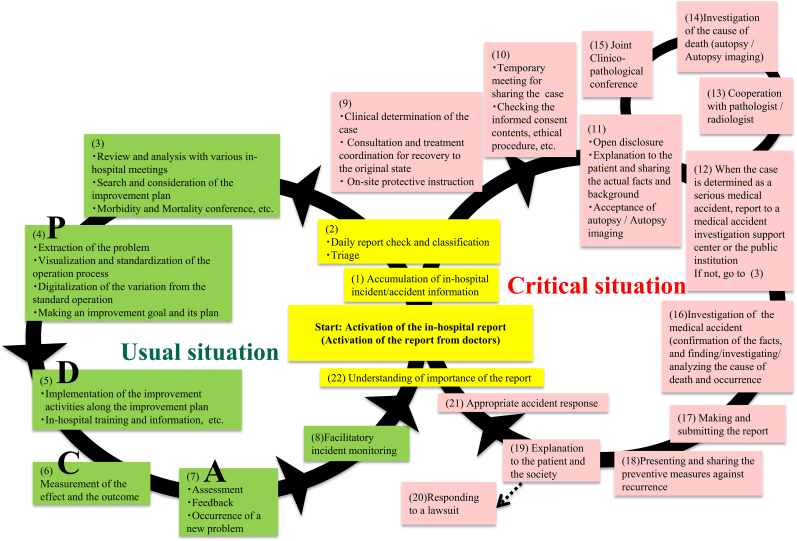
Loops of patient safety activities Thick black lines represent daily operation of patient safety. In usual situations with the Plan–Do–Check–Act cycle, patient safety operations are illustrated in the right loop, and in critical situations, in the left loop.

As a global turning point for patient safety, the 1990s were significant, and clarified patient safety managers’ comprehensive role in hospitals. Primarily, patient safety managers need to recognize the overall picture of patient safety, that is, safety in all and general medical services rather than just in their details; they should carefully analyze what aspects are missing in their hospitals and which equipment, facilities, and human resources need to be developed and deployed to fill the existing gaps. In this study, we divided patient safety management into usual and critical situations, illustrated it as loops (see Fig. 1), and outlined present safety problems and efforts.

## 1 Patient safety management during usual situations

In normal times, patient safety management includes the following: integration and triage of near-miss and hospital-wide comprehensive adverse event reports, analysis and extraction of these events' root causes, meetings with multi-professional healthcare providers, reviews of rules and manuals, consideration of measures to prevent recurrences; development of a contingency management plan, patient safety training and education, hospital-wide round monitoring, and quality improvement of all medical services. Plan–Do–Check–Act (PDCA) cycle, promoted by Deming [1]. as a learning and improvement characteristic, provide a widely accepted structure for improving the quality of a healthcare system [2]. Every day, institutional patient safety managers receive reports about several important issues and instruct the hospital staff about the required measures for improvement. However, they are unable to determine the number of staff actually practicing the suggested improvements, the results of the suggested improvements, or their effects in preventing serious medical accidents. Furthermore, medical institutions' prevention measures, descriptions, and data provide them little information about the effects of the patient safety efforts. During normal times in Japan, this crucial aspect is missing in the patient safety operations and is the reason the “P” (plan) of the PDCA cycle often remains a vague qualitative target, without concrete quantitative numerical goals. Indeed, quantitative numerical goals are difficult to establish, not because of patient safety managers' low capacity or inability but because, first, the occurrence of serious adverse events is rare compared with the total hospital-wide services. For a hospital, no one wants to establish a positive number as an outcome of serious adverse events (e.g., 1000-bed hospitals typically suffer zero to several fatal accidents a year). In addition, if an incident does not lead to a serious adverse event, it might not lead to an incident report, which is voluntary, either. Then, of course, the option to base outcomes on the number of incidents is not very helpful to patient safety management. As incident reports have been historically avoided as an outcome, their numerical reliability is low according to hospitals' populations. Therefore, only some event groups, such as falls, whose occurrence is relatively easy to grasp and whose absolute number is large, have been monitored as outcomes. Second, medical work processes are not standardized although the medical field has overcome similar issues using problem-solving methods such as “process standardization, measuring the deviation from the standard, and initiation of countermeasures.” In other words, one method is to establish a healthcare service procedure to produce reliable results, formulate various staff compliance trainings, and monitor the compliance rate and outcome process variations. Although such measurement is indirect, it can be applied to patient safety [3]. To our disappointment, myriad tasks are yet to be standardized, and hence, measuring compliance rates and variability is impossible even when researchers and practitioners desire to do so. In other words, from incident reports, safety management should identify hospital issues and establish stable medical service procedures for task categories because to reveal the latest achievement rates, safety managers must begin with baseline measurements. In addition, a certain period's variation should be evaluated by a controlled extent plan. For example, “increasing the implementation rate of the full name check to prevent patient misidentification accidents” is not an appropriate objective; the original plan states the following: In the procedure for preventing patient misidentification defined by the rules of a hospital, 60% of nurses and 40% of doctors currently perform collation between full name information from the patient and full name information in hand by medical staff; within one year, these percentages must rise to 90% for nurses and 70% for doctors. If any intervention plan is ambiguous, its effectiveness will remain unclear. In addition, if the intervention results remain unknown, the staff will be tired of the excessive rules required one after another and eventually fail to adhere to them. Contributing incident reports aimed at improvement will thus be disappointing, and the staff's reporting behavior will eventually fade. Furthermore, delayed reports will make it difficult to identify organizational issues, and in the event of an emergency (described in the next section), initiating an immediate response will be impossible. When serious adverse events occur within a vicious cycle, the staff will always react after the events, which then threaten to recur. Thus, patient safety managers must recognize the danger of a negative spiral. During normal situations, they must always pay attention to the following: quality control, instructions of the procedures of the multi-professional healthcare providers are checked in mutual co-operation, compliance with procedure rates, and measurement of available result variations. Reconsidering the institution's patient safety system from the perspective of required equipment, facilities, and human resources is crucial in a proper PDCA cycle, as is mutual support through partnerships.

## 2 Patient safety management during critical situations

When a serious issue occurs, medical institutions must quickly construct an emergency management system in an organized manner. During a crisis, operations to ensure patient safety include the following: cross-organizational treatment co-operation for patient recovery confirmed in each department; open disclosure to the patient; co-operation among the pathology and radiology departments to investigate a cause of death, if necessary; determining the necessity of notifying the medical accident investigation center and the police; medical accident investigation and report preparation; explanation of findings to patients; and publication of findings to society. Even during the normal times, patient safety management operations and personnel play an important role, but the hospital director has the pivotal role in making important decisions about responses to unusual situations. Indeed, initial response failure to a patient's serious safety issue sometimes threatens hospital closure. As serious accidents do not happen frequently, most hospital directors and patient safety personnel should recognize their unfamiliarity with handling a crisis. When facing the challenge of inconvenient facts, they often hesitate and then attempt to respond as optimistically as possible. This is largely congruent with “story generation” as a cognitive heuristic employed when attempting to understand a confusing situation. Hospital directors and chief patient safety officers must monitor the entire organization for story generation and make fair and objective situational decisions. In the short term, such decisions might be stressful for the hospital staff, partly out of fear of lacking objectivity and fairness. However, as not acting objectively and fairly might lead to a more serious situation for both the organization and the employee, it must be avoided. Hospital directors and chief patient safety officers must not lose sight of the mid-to long-term perspectives; they need to remember that fair behavior, independent of immediate interests, is the only way to protect the organizations, staff, and patients. In addition, they certainly need to pay attention to whether every hospital department reports serious issues responsibly. Medical doctors' reporting of incidents reflects organizational transparency and drives toward improvement in healthcare quality and safety. In addition, hospital-wide reporting of near-miss events is also significant because these events are precursors of adverse events. After identifying various clinical departments' high-risk areas, the next step should be to analyze the root cause of incidents, especially those reported by doctors, and intervene appropriately to improve healthcare quality [4]. This aspect should contribute directly to safer care and overall enforcement of the hospital's patient safety culture, particularly because reports from doctors are overwhelmingly more severe than those from other occupations; this means that hospitals cannot accurately ascertain adverse events unless doctors submit their reports. As chief safety officers, they want, as much as possible, to clear adverse events and respond to particularly serious adverse events by reporting the best about the hospital. However, as many medical institutions have indicated, physicians are still reluctant to report accidents. If safety managers believe the reporting of serious accidents is weak, hospital administration should consider activating physicians' reporting behavior or tasking the patient safety department to introduce a required screening and reporting system for accidental cases of death. In a crisis, one of the most important tasks is co-ordination of treatment in medical accident cases. For example, if a drug overdose occurs, that fact should be immediately reported to the safety management department, and experts from multiple departments should gather to measure the drug's blood concentration, administer an antagonist, remove the drug by plasma exchange, and pool all resources to co-ordinate life-saving treatment. Many patient deaths result from medical accidents caused by delays, missing reports, or lack of appropriate initial responses. Certainly, co-ordination of treatment requires resilience and flexible responses. The hospital director must assist the safety management department by ensuring that on-site reporting and co-ordination is conducted. “Open disclosure” is a further requirement, that is, open discussion with the patient, their families, their caregivers, and other support persons about incidents that result in harm to a patient receiving healthcare. In other words, open disclosure expresses apology and compassion for the patient having received irregular healthcare. Hospital staff should promptly explain the facts currently grasped about the situation and examine ways in which undiscovered facts could be grasped. However, the timing of disclosure can be crucial. Immediate disclosure after an accident might cause cognitive discrepancy between the patient and hospital staff, leading to a critical gap in communication. The hospital management board should monitor whether open disclosure is appropriately conducted. Deciding whether to apply the Japanese medical accident investigation system is also an important decision that the hospital director has to make [5]. To avoid ad hoc responses, supporting the organization with ingenuity can maintain the objectivity and transparency of the decision-making process and clarify the basis for decisions. The hospital director and the chief safety officer need a mutually supportive partnership in normal situations and strong leadership in crises. The most important actions are to ensure the following: patient safety, accurate fact-finding, open disclosure, verification of cause analysis, and recurrence prevention. The usual and the unusual or critical are linked like loops. Hence, the hospital director and the chief safety officer should be conscious of linked activities, have consistent attitudes, and make fair and objective decisions in both crises and normal situations. Overall, healthcare quality improvement must be continuous, and seamless efforts toward patient safety should continue apace.

## 3 Loops of patient safety activities

We described the comprehensive double loop activities for patient safety managements. The loop described as patient safety management during critical situations on right side and patient safety management during usual situations on left side and is represented as a continuous infinity (∞) shape (Fig. 1). Either way, the starting point is activation of an in-hospital report. Especially, activation of reports from doctors. Significance of medical doctors’ incident reports for organizational transparency and as a driving force for patient safety. ^4^ We have placed the starting point for this double loop figure here as point zero. We created a cyclical model in which these activities are properly carried out, the importance of reporting is understood, and it returns to activation of in-hospital reporting.

The contents of usual situations on left side:

(1) Accumulation of in-hospital incident/accident information. Patient safety incident reporting is mandatory for all staff.
(2) Daily report checks and classification. Triage. Follow-up or quick response. Triage at various in-hospital meetings.
(3) Review and analysis at various in-hospital meetings. Search and consideration of improvement plan. Morbidity and mortality conferences. Objectives of the improvement plan, including morbidity and mortality conferences, are to identify adverse outcomes associated with medical accidents, modify behavior and judgment based on previous experiences, and prevent repetition of errors leading to complications.
(4) Extraction of the problem. Visualization and standardization of the operation process. Digitalization of variation from standard operation. Creating an improvement goal and its plan. Planning process for quality improvement.
(5) Implementation of the plan's improvement activities. In-hospital training and information. Practicing the quality improvement process.
(6) Measurement of the effect and outcome. Checking the quality improvement process.
(7) Assessment. Feedback. Occurrence of a new problem. Enacting the quality improvement process again.
(8) Facilitatory incident monitoring. Standardization and improvement reporting culture.

The contents of usual critical situations on right side:

(9) Clinical determination of the case. Consultation and treatment co-ordination for recovery to the original state. On-site protective instruction. Cross-organizational treatment co-operation for patient recovery.
(10) Meetings for sharing cases. Checking the informed consent content and ethical procedures. Confirmation of proper informed consent.
(11) Open disclosure. Explanation to the patient and sharing facts and background. Acceptance of autopsy/autopsy imaging. Each department's confirmation of facts. In case of death, explain the necessity of autopsy and autopsy imaging for the cause-of-death investigation to the bereaved family.
(12) When the case is determined a serious medical accident, report to a medical accident investigation support center and public institution. If not, go to No. 3. Determining the necessity of notifying the medical accident investigation center and the police, thereby demonstrating organizational transparency. If the case does not fall under the medical accident investigation system, it is allocated for review and analysis at various in-hospital meetings. Search and consideration of the improvement plan.
(13) Co-operation with pathologist/radiologist. Search for the cause of death.
(14) Investigation of the cause of death (autopsy/autopsy imaging). Search for the cause of death.
(15) Joint clinicopathological conference. Search for the cause of death.
(16) Investigation of the medical accident (confirmation of facts and finding/investigating/analyzing the cause of death and its occurrence). Identify a new basic idea of investigation in medical accidents, which is to clarify the cause of the accident and link the findings for prevention of recurring accidents.
(17) Writing and submitting the report. Summarize the cause of the accident and suggest how findings can prevent recurring accidents.
(18) Presenting and sharing preventive measures against recurrence. Share the point of issue for healthcare providers.
(19) Explanation to the patient and society. Responsibility as a part of social infrastructure.
(20) Responding to a lawsuit. Conference for reconciliation with the patient and the bereaved family.
(21) Appropriate accident response. Growth of organizational safety culture through accumulation of appropriate responses.
(22) Understanding the incident report's importance. Finally, both in usual and critical times, incident reports are important for organizational transparency and driving forces for patient safety.

## 4 Conclusion

Advanced healthcare is actually on the dangerous foundation of basic patient safety and infection control. Trust was piled up, in one big accident will be destroyed. Many efforts have been made to patient safety, but the current situation is that the pattern is weak and the results are not clear. We work on and practice patient safety as a model that links patient safety and improves the quality of patient care. By creating a loop diagram, the overall picture of patient safety work (usual and critical situation) was grasped. The loop diagram was considered to be an important and useful tool for evaluating the patient safety system. What is important in this type is the initial response to an emergency, which is the first corner of the light loop. It is patient safety that we practice to emphasize the initial response to an emergency, create a mold that does not shake, and take measures without omissions.

Although the concept of patient safety has grown and spread rapidly worldwide, it still seems merely added onto existing medical systems. We thus need to shift our paradigm to patient safety-centered healthcare, in which clinical governance is a fundamental concept. The United Kingdom's National Health Service (NHS) defines clinical governance as a framework through which NHS organizations are accountable for continually improving their service quality and safeguarding high standards of care by creating an environment in which excellent clinical care flourishes [6,7]. In other words, the NHS has systematically approached the maintenance and improvement of patient-care quality. Finally, frequent medical accidents might be related to medical progress because medical advances have created complex processes and team healthcare, requiring control of potentially large numbers of human and communication errors [8]. Medical care will be an acquired social value that is provided safely, and uncontrolled medical error can also sometimes be a dangerous weapon to the patient. Thus, all medical care and practice demands the concept of patient safety. The WHO Patient Safety Curriculum Guide describes patient safety in both developed and developing countries as a broad subject: it can incorporate the latest technology but still requires washing hands correctly and knowing how to be an effective team member.3 Many features of patient safety programs do not involve financial resources but rather individuals' commitment to practice safely. All persons involved in medical care need to be conscious of safety and act accordingly. Patient safety managers require determination, belief, and leadership.

In this way, we have learned and think that expanding the wearable molds nationwide will contribute to the improvement of patient safety, and welfare research on the training of doctors who have expertise in patient safety and the measurement of risk at patient institutions. We are working as a labor research institute. The “CQSO Project for Chief Quality Safety Officers” is being developed at Nagoya University to train doctors who specialize in patient safety. With the support of the Japanese Ministry of Education, Culture, Sports, Science, and Technology for 5 years from 2014. It was held as ASUISHI project. From 2019, the project was supported by the Ministry of Health, Labor, and Welfare. Renewal points are Training leader doctors specializing in patient safety and introducing Toyota's quality control method to patient care Develop students' problem-solving skills. Those are the two major pillars.

## Availability of data and materials

All data generated or analyzed during this study are included in this published article.

## Competing interests

The authors declare that they have no competing interests.

## Ethical approval

In order to keep the ethical soundness of the research, an ethical approval letter was obtained from the Institutional Review Board (IRB) of Nagoya University Hospital. Comprehensive consent was also secured before data collection. (No. 2018–0283).

## Sources of funding for your research

This work was supported by 10.13039/501100001691JSPS KAKENHI, Grant Number 201620007B for YN and 10.13039/501100008645Terumo Life Science Foundation (Kanagawa, Japan) for TF.

## Author contribution

TF and YN interpreted the data, drafted the manuscript, and revised the manuscript for important intellectual content. TF drafted the manuscript and revised the manuscript for important intellectual content. YN contributed to the acquisition of data, conducted data cleaning, and interpreted the data. YN also conceived and designed this study, interpreted the data, and revised the manuscript for important intellectual content. T.F. wrote the main manuscript text and Y.N. prepared the figure. All authors reviewed the manuscript. All author have approved the final manuscript.

## Registration of research studies

Name of the registry:
Nagoya University Hospital
Unique Identifying number or registration ID:
2018–0283
Hyperlink to your specific registration (must be publicly accessible and will be checked):
https://www.med.nagoya-u.ac.jp/medical_J/ethics/

## Consent

We confirmed the consent.

## Guarantor

Tatsuya Fukami.

## Declaration of competing interest

The authors state that they have no Conflict of Interest.
